# Catastrophic Acute Aortic Dissection in a Young Healthy Patient

**DOI:** 10.1016/j.jaccas.2025.105230

**Published:** 2025-09-17

**Authors:** Mohammad Abdallah Omar, Ahmad Alkhatib, Simardeep Singh, Rahmeh Alasmar, Tareq Hanouneh, Ishaque Hameed, Pankti Desai

**Affiliations:** a MedStar Health Georgetown University (Baltimore) Program, Baltimore, Maryland, USA; b University of Jordan Faculty of Medicine, Amman, Jordan

**Keywords:** aorta, chest pain, dissection, genetics, hypertension, vascular disease

## Abstract

**Background:**

Acute type A aortic dissection (ATAAD) typically affects older patients with hypertension or connective tissue disorders; occurrence in young adults without identifiable risk factors is rare and diagnostically challenging.

**Case Summary:**

A healthy 41-year-old man presented with sudden severe chest pain, markedly elevated blood pressure, and tachycardia. Computed tomography angiography revealed extensive ATAAD involving multiple major vessels. Emergency surgery, including aortic root replacement and frozen elephant trunk repair, was complicated by severe postoperative coagulopathy, end-organ failure, and brain death.

**Discussion:**

Approximately 30% of ATAAD cases are initially misdiagnosed, particularly in young patients with atypical presentations. This case highlights the importance of diagnostic suspicion even without traditional risk factors, and it reinforces recent guidelines from the American College of Cardiology/American Heart Association recommending screening aortic imaging and genetic counseling of first-degree relatives, aiding preventive management.

**Take-Home Messages:**

It is important to maintain high suspicion for ATAAD in young patients. Rapid imaging, immediate surgical intervention, and family screening are essential.

## Graphical Abstract

**Figure:**
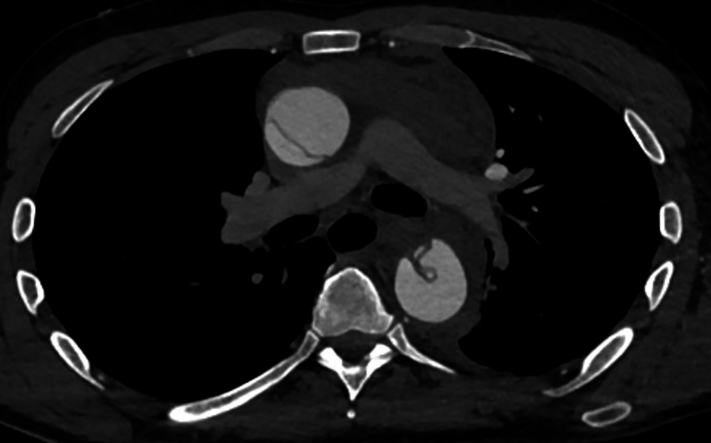

## History of Presentation

A 41-year-old man with no significant medical history presented to the emergency department with acute, severe, tearing retrosternal chest pain radiating to his back and associated with diaphoresis, nausea, and anxiety. He denied syncope, dyspnea, or neurological symptoms. Vital signs included markedly elevated blood pressure (200/100 mm Hg) and tachycardia (110 beats/min). Examination showed symmetric pulses, with no cardiac murmurs, neurological deficits, or signs of connective tissue disease.

**Take-Home Messages**

• Maintain diagnostic vigilance: Acute aortic dissection should be suspected even in young, seemingly low-risk individuals with acute severe chest or back pain.
• Consider genetic counseling: Even without obvious syndromic features, genetic screening is crucial in ATAAD patients to guide preventive care for their families.
• Rapid response saves lives: Mortality risk increases dramatically (1%-2% per hour) with delays; hence, immediate imaging and surgical intervention are paramount.

## Past Medical History

The patient had no prior medical conditions, surgical history, or substance use. He was a nonsmoker, and he had no family history of cardiovascular diseases or sudden cardiac death, with no diagnosis of hypertension or connective tissue disorders.

## Differential Diagnoses

The patient presented with red flag features that warranted urgent attention. The acuity of the chest pain along with its tearing nature, radiation to the back, severity, and markedly elevated blood pressure suggested an aortic etiology of chest pain. The absence of established risk factors for aortic disease, such as past medical history of hypertension, illicit substance use, family history of heritable thoracic aortic disease (HTAD), and gross features of connective tissue diseases, necessitated further investigation into other causes of acute chest pain, including acute coronary syndrome, pneumothorax, rib fractures, pulmonary embolism, acute pericarditis, and musculoskeletal chest pain.

## Investigations

**Figure 1:**
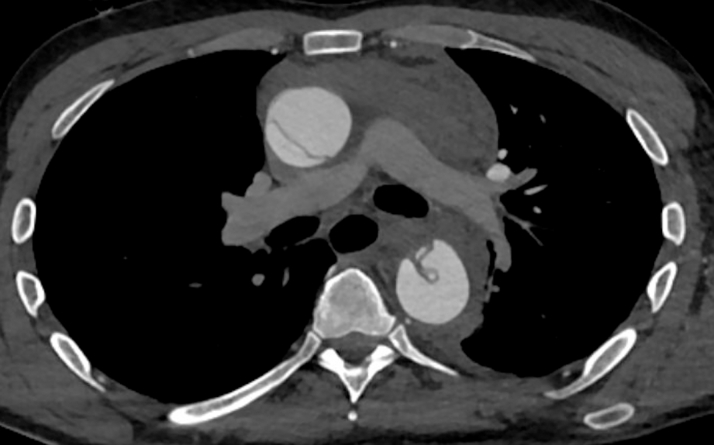
Axial Computed Tomography Demonstrating Acute Dissection of the Ascending and Descending Aorta

**Figure 2:**
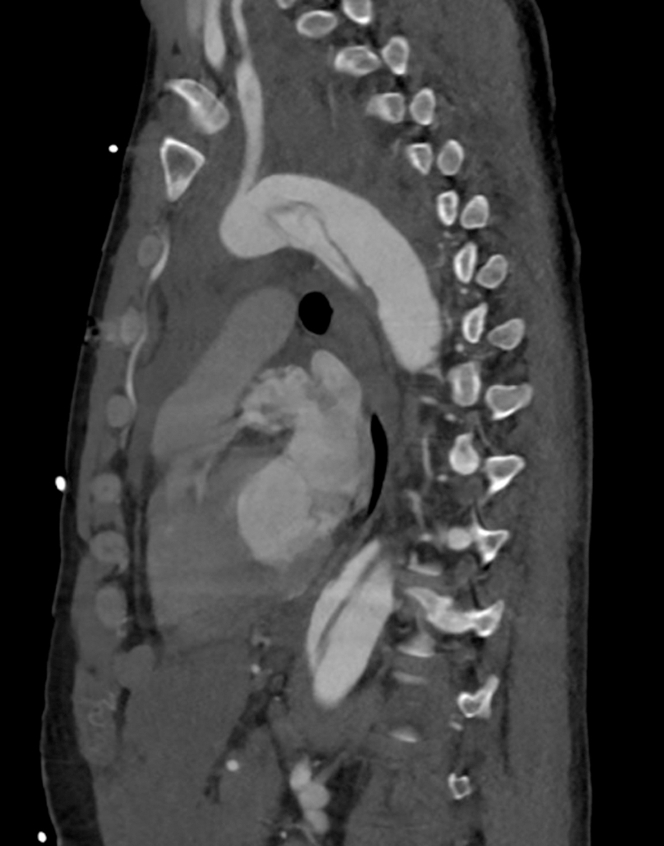
Sagittal Computed Tomography Demonstrating the Extension of Acute Dissection of the Aorta From the Aortic Arch Down to the Descending Aorta

**Figure 3:**
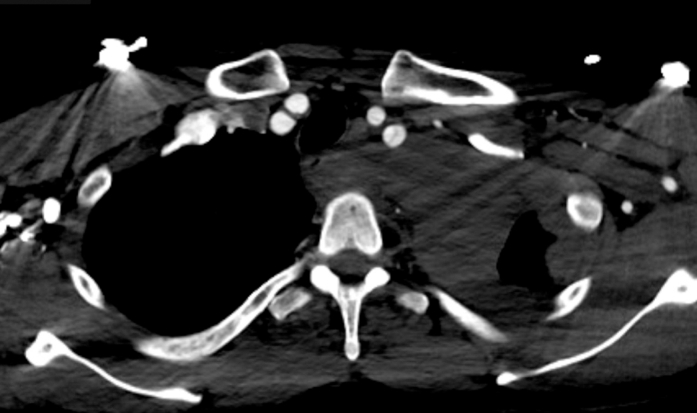
Axial Computed Tomography Demonstrating Acute Dissection of the Left Subclavian and Left Common Carotid Arteries

**Figure 4:**
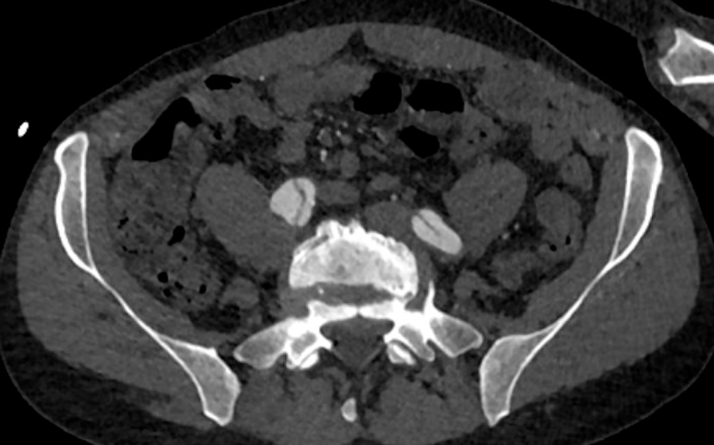
Axial Computed Tomography Demonstrating the Extent of Acute Dissection to the Iliac Arteries

Initial electrocardiogram revealed sinus tachycardia with nonspecific changes. Cardiac biomarkers were negative (troponin-I undetectable). D-dimer was elevated (2,500 ng/mL), and chest radiograph demonstrated a widened mediastinum. Urine toxicology was negative for illicit substance use. Computed tomography (CT) angiography identified an extensive acute type A aortic dissection (ATAAD) extending from the sinotubular junction through the common iliac bifurcation, involving major branches such as the brachiocephalic, carotid, subclavian, celiac trunk, superior mesenteric, renal, and iliac arteries. Moreover, focal rupture of the false lumen was observed at the proximal descending aorta, with extensive mediastinal and pericardial hemorrhage, and an ascending aortic aneurysm (5.4 cm) causing compression of the left atrium (Figures 1, 2, 3 and 4).

## Management

**Figure 5:**
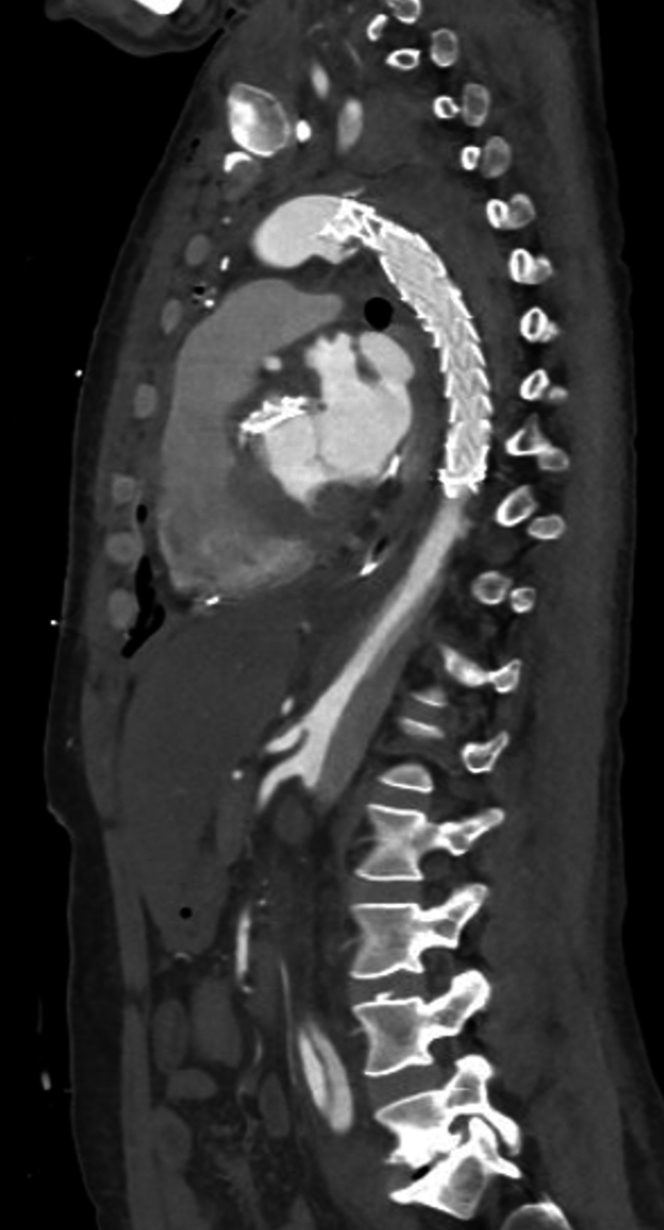
Sagittal Computed Tomography Showing the Aorta After Aortic Root Replacement Surgery

An esmolol drip was started for blood pressure and heart rate control. Intravenous morphine was administered for pain control. The patient was emergently taken to the operating room. Emergency surgery involving aortic root replacement, total arch replacement, and frozen elephant trunk graft placement was performed under circulatory arrest with antegrade cerebral perfusion (Figure 5).

The aortic arch was transected, and the supra-aortic branches were excised en bloc and reimplanted as an island onto a Gelweave graft (Terumo), which was anastomosed to the descending thoracic aorta. A thoracic endovascular aortic repair graft was also deployed in antegrade fashion via the surgical field to reinforce the descending aorta. The coronary buttons were reimplanted into the valve-conduit, and the patient was successfully weaned from cardiopulmonary bypass. Because of friable tissues and concern for postoperative bleeding, the chest was temporarily left open and packed, with a wound vacuum device placed for delayed closure.

## Outcome and Follow-Up

The postoperative course was complicated by severe coagulopathy, end-organ failure, and refractory bleeding, necessitating extensive blood product transfusion and continuous renal replacement therapy. The following day, the patient was taken again to the operating room for mediastinal washout and sternotomy incision closure. Subsequently, the patient exhibited progressive neurological decline. CT confirmed cerebral edema and herniation, and brain death was declared the following day.

Considering his young age, ATAAD diagnosis, and thoracic aneurysm size, HTAD screening was initiated in accordance with the 2022 American College of Cardiology (ACC)/American Heart Association (AHA) and European Society of Cardiology guidelines. First-degree relatives underwent imaging, which initially showed no abnormalities; however, genetic screening and regular surveillance were advised.

## Discussion

Although ATAAD predominantly affects older individuals, approximately 7% of cases occur in individuals younger than 40 years, often associated with hypertension, genetic syndromes, illicit substance use, or congenital anomalies such as bicuspid aortic valves.^1^, ^2^, ^3^ This case of a previously healthy 41-year-old man, who lacked identifiable risk factors or clinical features suggestive of a heritable condition, underscores 2 important clinical considerations: first, the possibility of ATAAD even in younger patients without traditional cardiovascular risk factors, and second, the rapid and catastrophic nature of this condition despite timely diagnosis and surgical intervention.

The rarity of ATAAD in young adults without risk factors poses diagnostic challenges, often delaying diagnosis and intervention. Approximately 30% of ATAAD cases are initially misdiagnosed as acute coronary syndrome, musculoskeletal pain, or pulmonary embolism, particularly when classic presentations (eg, tearing chest or back pain, pulse deficits) are less pronounced or absent.^4^^,^^5^ In our patient, rapid clinical suspicion and early imaging led to timely recognition, reinforcing the critical role of high diagnostic vigilance.

In this case, the patient also had a markedly elevated D-dimer. Although D-dimer is a nonspecific marker, it is often elevated in acute aortic dissection owing to activation of coagulation and fibrinolysis triggered by disruption of the aortic wall. According to the ACC and AHA, while no single biomarker confirms the diagnosis of aortic dissection, a D-dimer level of <500 ng/mL in a patient with low pretest probability can help exclude acute aortic syndromes.^6^ Conversely, in this case, the elevated D-dimer level supported a higher index of suspicion, especially in the context of tearing chest pain with radiation to the back. This underscores the utility of integrating clinical risk scoring with biomarker assessment in the early triage of patients with potential acute aortic syndrome, even among younger individuals without known cardiovascular risk factors.

CT angiography remains the first-line imaging modality in diagnosing ATAAD. It is widely available and offers near-perfect sensitivity and specificity (≥98%) for identifying intimal tears and dissection extent.^6^, ^7^, ^8^ Alternative methods, such as transesophageal echocardiography, are particularly beneficial in hemodynamically unstable patients given its bedside availability and real-time diagnostic capability for identifying complications such as tamponade or acute aortic regurgitation.^6^^,^^7^ In this case, CT angiography clearly demonstrated the dissection, enabling rapid surgical intervention. Despite timely diagnosis and operative management, the patient experienced rapid hemodynamic collapse, reflecting the aggressive progression that can occur even in the setting of optimal care.

Pathological evaluation provided critical insight into underlying mechanisms. Gross examination of the resected aortic segment revealed severe disruption without evidence of atherosclerosis or calcification. Microscopically, the aortic wall showed marked myxoid degenerative changes, patchy smooth muscle nuclear loss, and extensive intramural and adventitial hemorrhage. Such findings are consistent with cystic medial degeneration, a hallmark of HTAD, even in the absence of overt syndromic features such as Marfan or Loeys-Dietz syndrome.^6^^,^^9^ Additionally, examination of the patient's structurally normal tricuspid aortic valve showed subtle myxoid degeneration and focal fibrosis, further supporting connective tissue abnormality at the histological level.

These pathology findings underscore the critical importance of genetic counseling and testing, as recommended by the 2022 ACC/AHA guidelines for aortic disease, for patients presenting with aortic root/ascending aortic aneurysms or aortic dissection who have risk factors for HTAD, such as a family history of thoracic aortic disease, syndromic features (eg, Marfan or Loeys-Dietz syndrome), or early age of onset.^6^ Genetic testing can identify pathogenic variants in genes such as *ACTA2*, *MYH11*, and *TGFB2*, potentially altering surveillance strategies and intervention thresholds for the patient's first-degree relatives.^6^^,^^9^ Although postmortem genetic analysis was not conducted here owing to clinical circumstances, such testing should be strongly considered in similar cases to inform risk assessment and preventive management in at-risk family members.

**Visual Summary:** Timeline of Clinical Events

Time	Clinical Events and Management
Day 1, 07:00	41-year-old previously healthy man presented with sudden severe tearing retrosternal chest pain radiating to his back with blood pressure of (200/100 mm Hg) and tachycardia (110 beats/min). Chest radiograph revealed widened mediastinum.
Day 1, 08:00-09:00	Urgent computed tomography angiography confirmed extensive acute type A aortic dissection from sinotubular junction to common iliac arteries, a 5.4-cm ascending aneurysm. Emergency surgical team activated.
Day 1, 09:30-16:30	Underwent emergency surgical repair, including aortic root replacement and frozen elephant trunk procedure under deep hypothermic circulatory arrest with antegrade cerebral perfusion. Notable coagulopathy observed.
Day 1, 16:30-Day 2, 06:00	Severe postoperative coagulopathy end-organ failure requiring massive transfusions, and initiation of continuous renal replacement therapy owing to acute renal failure.
Day 2, 06:00-18:00	Persistent metabolic acidosis, end-organ failure, progressive hemodynamic instability. Mediastinal washout and sternotomy incision closure. Worsening neurological status observed. Emergency imaging confirmed severe cerebral edema and herniation.
Day 3, 08:00-10:00	Brain death confirmed.

## Conclusions

ATAAD remains a rapidly fatal condition that may occur unpredictably in young, healthy individuals. This case highlights the essential roles of early suspicion, rapid imaging, emergency surgical management, and genetic screening to optimize outcomes and preventive strategies in thoracic aortic disease.

## Funding Support and Author Disclosures

The authors have reported that they have no relationships relevant to the contents of this paper to disclose.
